# Investigation of Electromechanical Characteristics of Piezoelectric Unimorph Actuators Under Thermal-Vacuum Conditions Relevant to Low Earth Orbit

**DOI:** 10.3390/mi17091038

**Published:** 2026-08-30

**Authors:** Laurynas Šišovas, Linas Juknevičius, Andrius Čeponis

**Affiliations:** 1 Department of Aeronautical Engineering, Antanas Gustaitis Aviation Institute, Vilnius Gediminas Technical University, Linkmenų Str. 28-4, LT-08217 Vilnius, Lithuania; 2 Antanas Gustaitis Aviation Institute, Vilnius Gediminas Technical University, Linkmenų Str. 28-4, LT-08217 Vilnius, Lithuania; 3 Institute of Mechanical Science, Faculty of Mechanics, Vilnius Gediminas Technical University, Plytinės Str. 25, LT-10105 Vilnius, Lithuania

**Keywords:** piezo, unimorph, space, adhesive, vacuum, LEO

## Abstract

Piezoelectric unimorph actuators are promising candidates for precision actuation systems in small satellites, where their electromechanical performance must remain predictable under vacuum and temperature variations. However, actuator performance is determined not only by the piezoelectric material but also by the passive layer and bonding interface, whose combined influence under thermal-vacuum conditions requires systematic evaluation. This study presents a numerical and experimental investigation of the temperature-dependent electromechanical characteristics of piezoelectric unimorph actuators incorporating PIC 181 piezoceramic plates, passive layers made of 7075-T6 aluminum or Ti-6Al-4V titanium, and three different vacuum-compatible bonding materials. Numerical analyses were performed to determine modal, impedance–frequency, and displacement–frequency characteristics over the temperature range of 253.15–323.15 K, while experimental investigations under vacuum conditions quantified the additional influence of the bonding interface. Increasing temperature caused a decrease in resonance frequency for both passive-layer configurations. Actuators with Ti-6Al-4V passive layers generally exhibited higher resonance frequencies, lower impedance values, and larger displacement amplitudes than those with 7075-T6 aluminum passive layers. The maximum measured displacement amplitudes reached 23.11 µm for the aluminum-based actuator and 29.67 µm for the titanium-based actuator at 323.15 K. Among the investigated bonding materials, BM No. 3 consistently provided the lowest impedance and highest displacement response. The results demonstrate that passive-layer and bonding-material selection should be considered jointly and identify the Ti-6Al-4V/BM No. 3 combination as the most favorable among the investigated configurations for resonant piezoelectric unimorph actuation under the tested thermal-vacuum conditions.

## 1. Introduction

Piezoelectric actuators are widely used in precision mechatronic systems due to their high resolution, fast dynamic response, compact dimensions, low power demand and ability to generate controllable motion without complex transmission mechanisms [1,2,3]. These characteristics make them particularly attractive for aerospace and space applications, where mass, volume, reliability and positioning accuracy are among the most critical design constraints [4,5]. In small satellites, piezoelectric actuation can be applied in optical alignment systems, beam-steering mechanisms, vibration control devices, micro-positioning platforms and adaptive structural elements. In such systems, piezoelectric unimorph actuators can be integrated as compact actuation elements to provide fine positioning, angular adjustment, vibration suppression, or controlled deformation of optical and structural components. These functions are particularly relevant to small satellite applications where low mass, compact dimensions, low power consumption, and precise motion control are required. [6,7,8]. For such applications, actuator performance must remain predictable not only under laboratory conditions, but also under environmental conditions representative of orbital operation [9,10].

Low Earth Orbit represents a demanding operational environment for precision electromechanical systems. Components operating in this environment are exposed to vacuum, cyclic temperature variation and thermally induced stresses caused by repeated transitions between solar illumination and eclipse [11,12,13]. These environmental factors may affect the mechanical stiffness, dielectric properties, damping behavior and electromechanical coupling of piezoelectric structures [14,15]. Since many piezoelectric actuators are operated close to their resonance frequencies in order to achieve high displacement amplitudes and efficient energy conversion, even moderate temperature-induced shifts in resonance frequency, impedance, or displacement response can reduce operational stability and control accuracy of the whole system [16,17]. In the context of the present study, LEO-relevant conditions refer specifically to the thermal-vacuum environment, characterized by high vacuum and temperature variations associated with orbital operation. The present investigation therefore focuses on the thermal-vacuum component of the LEO environment, while other environmental factors associated with orbital operation are outside the scope of this study.

Among different piezoelectric actuator configurations, unimorph actuators are one of the most structurally simple and efficient solutions for generating bending motion [18,19]. A typical unimorph actuator consists of an active piezoelectric layer bonded to a passive elastic layer. When an electric field is applied, the piezoelectric layer undergoes deformation, while the passive layer constrains this deformation and converts the induced strain mismatch into bending displacement [20,21]. Therefore, the electromechanical response of a unimorph actuator is determined not only by the properties of the piezoceramic material, but also by the passive-layer material, bonding interface, clamping conditions and thermal compatibility between the joined layers [22,23].

The passive layer plays a significant role in defining the stiffness-to-mass ratio, resonance frequency and displacement amplitude of the actuator [24]. Materials such as aluminum alloys and titanium alloys provide different combinations of density, stiffness and thermal response behavior [25,26]. Aluminum alloys offer low density and good manufacturability, whereas titanium alloys provide higher stiffness, improved mechanical stability and lower thermal response compared with aluminum [27,28,29]. As a result, the selection of the passive layer material directly influences the dynamic behavior of the unimorph actuator, particularly under temperature-variable conditions.

In addition to the passive layer, the bonding layer is a critical element of the unimorph structure [30,31]. The adhesive layer ensures mechanical coupling between the piezoceramic and passive layer and is responsible for effective strain transfer across the interface. Its stiffness, damping capacity, curing quality, thickness stability and thermal behavior can significantly affect resonance and impedance characteristics as well as displacement amplitudes [32,33]. Under thermal-vacuum conditions, the bonded interface may experience additional stresses due to mismatch in thermal expansion coefficients between the piezoceramic, adhesive and passive layer. Consequently, the bonding material cannot be treated merely as a technological joining medium, because it becomes an active structural factor influencing the overall electromechanical response of the actuator.

Numerical modeling is commonly used to predict the modal behavior, impedance–frequency response and displacement characteristics of piezoelectric actuators [34]. However, idealized numerical models cannot fully represent manufacturing-related factors such as adhesive curing conditions, surface preparation, local bonding imperfections, residual stresses and thermal-vacuum effects on the adhesive interface [35]. Therefore, experimental assessment remains essential for validating actuator behavior and for identifying the combined influence of passive-layer material, bonding material and temperature on electromechanical performance.

Although previous studies have extensively investigated the structural design and dynamic behavior of piezoelectric unimorph actuators, the effects of passive-layer properties, adhesive bonding and temperature have generally been considered separately. In particular, systematic experimental data describing how the passive metallic layer and bonding interface jointly affect the resonant electromechanical behavior of unimorph actuators under vacuum and temperature conditions relevant to space applications remain limited. This is important because the bonding layer contributes to interfacial stiffness, damping and thermally induced stresses, while the passive layer determines the global stiffness-to-mass ratio of the actuator. Consequently, their combined influence may significantly affect resonance frequency, electrical impedance and vibration amplitude as temperature varies.

The novelty of this study therefore lies in a comparative numerical–experimental assessment of piezoelectric unimorph actuators combining two aerospace-relevant passive-layer materials, 7075-T6 aluminum and Ti-6Al-4V titanium, with three different vacuum-compatible bonding materials over the temperature range of 253.15–323.15 K under vacuum conditions. The numerical investigation establishes the temperature-dependent response associated with the passive-layer configurations without introducing adhesive-related effects, while the experimental investigation subsequently quantifies the influence of the bonding interface. Resonance frequency, impedance at resonance and out-of-plane displacement are evaluated using identical actuator geometry, excitation and clamping conditions. This approach allows the contributions of passive-layer selection and bonding material to be distinguished and provides experimentally supported criteria for selecting unimorph material combinations for thermally variable vacuum environments.

## 2. Structural Design of Piezoelectric Unimorph Actuators

**Figure 1:**
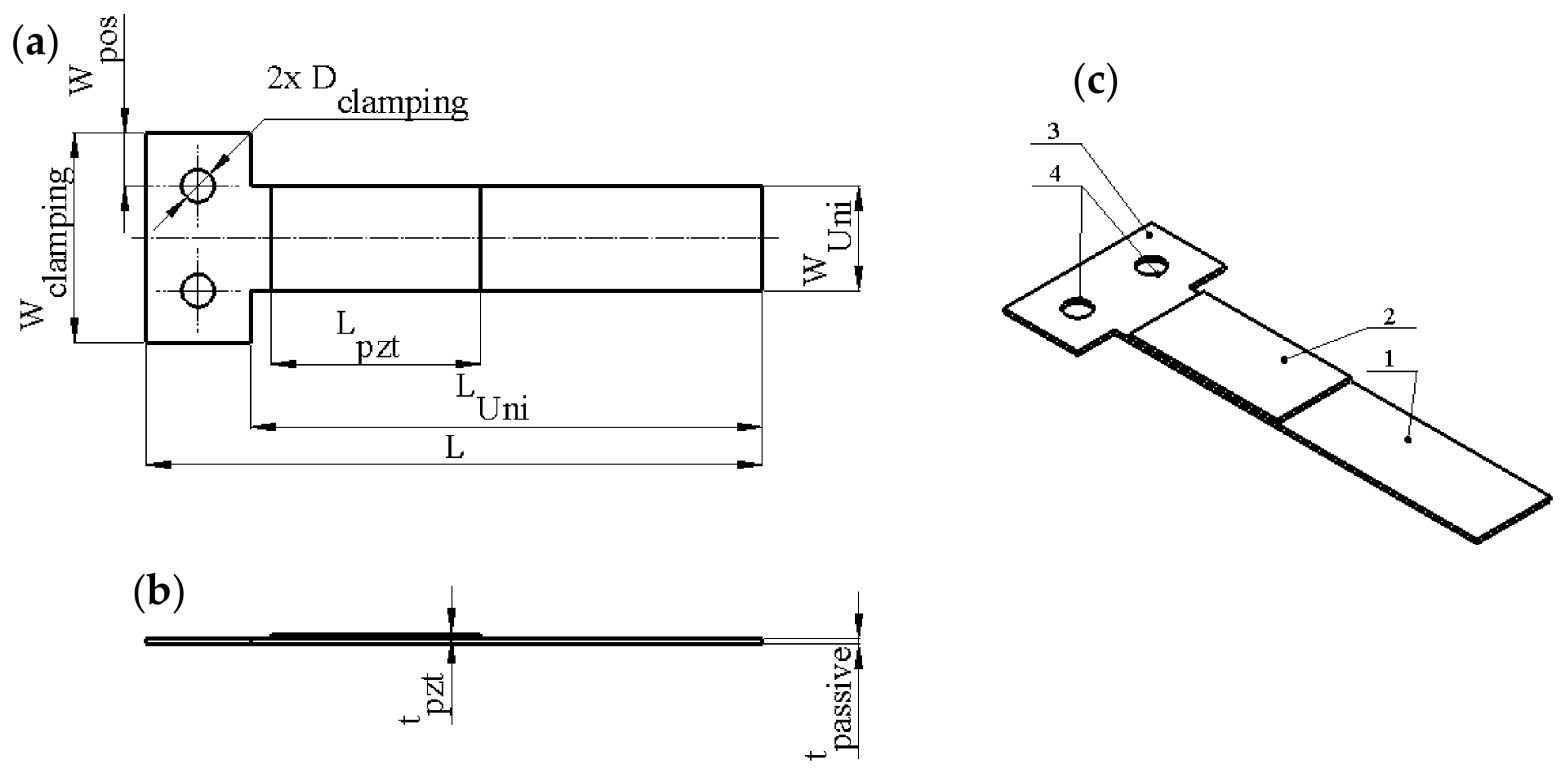
Structural design of unimorph actuator (**a**)—top view; (**b**)—side view; (**c**)—isometric view; 1—passive layer; 2—piezoelectric layer; 3—rectangular clamping zone; 4—clamping holes.

The piezoelectric actuator was designed as a unimorph bending structure composed of one active piezoelectric layer and one passive elastic layer. The active layer was made of PIC 181 piezoceramic material, whereas two passive-layer configurations were considered, i.e., 7075-T6 aluminum alloy and Ti-6Al-4V titanium alloy. The passive metallic layer provides mechanical support to the piezoelectric plate and defines the overall stiffness, mass distribution and dynamic response of the actuator. When an electric field is applied to the active piezoelectric layer, the strain mismatch between the active and passive layers produces out-of-plane bending motion of the unimorph structure. The structural design of the investigated piezoelectric unimorph actuator is presented in Figure 1.

The geometry of the actuator was selected to provide a clearly defined bending response and repeatable mechanical fixation during numerical and experimental investigations. The actuator consists of a free bending section and an enlarged rectangular clamping zone. The piezoelectric plate is bonded to the passive layer close to the clamping region, where strain transfer between the active and passive layers is important for the generation of controlled bending motion. The rectangular clamping zone provides stable fixation to the support structure and enables a well-defined boundary condition during modal, impedance and displacement measurements. Two clamping holes were incorporated into this region to allow mechanical fastening of the actuator while maintaining alignment and reducing unwanted variations in the mounting conditions. The main geometrical parameters of the actuator are summarized in Table 1.

The total actuator length was 55 mm, while the free, unclamped length was 45 mm. The width of the unimorph section was 10 mm, and the width of the rectangular clamping zone was 20 mm. The active piezoelectric layer had a length of 20 mm. Both the piezoelectric and passive layers had a thickness of 0.5 mm. Such dimensions were selected to obtain a compact bending actuator with a measurable second out-of-plane bending mode within the frequency range suitable for numerical and experimental electromechanical analysis.

**Figure 2:**
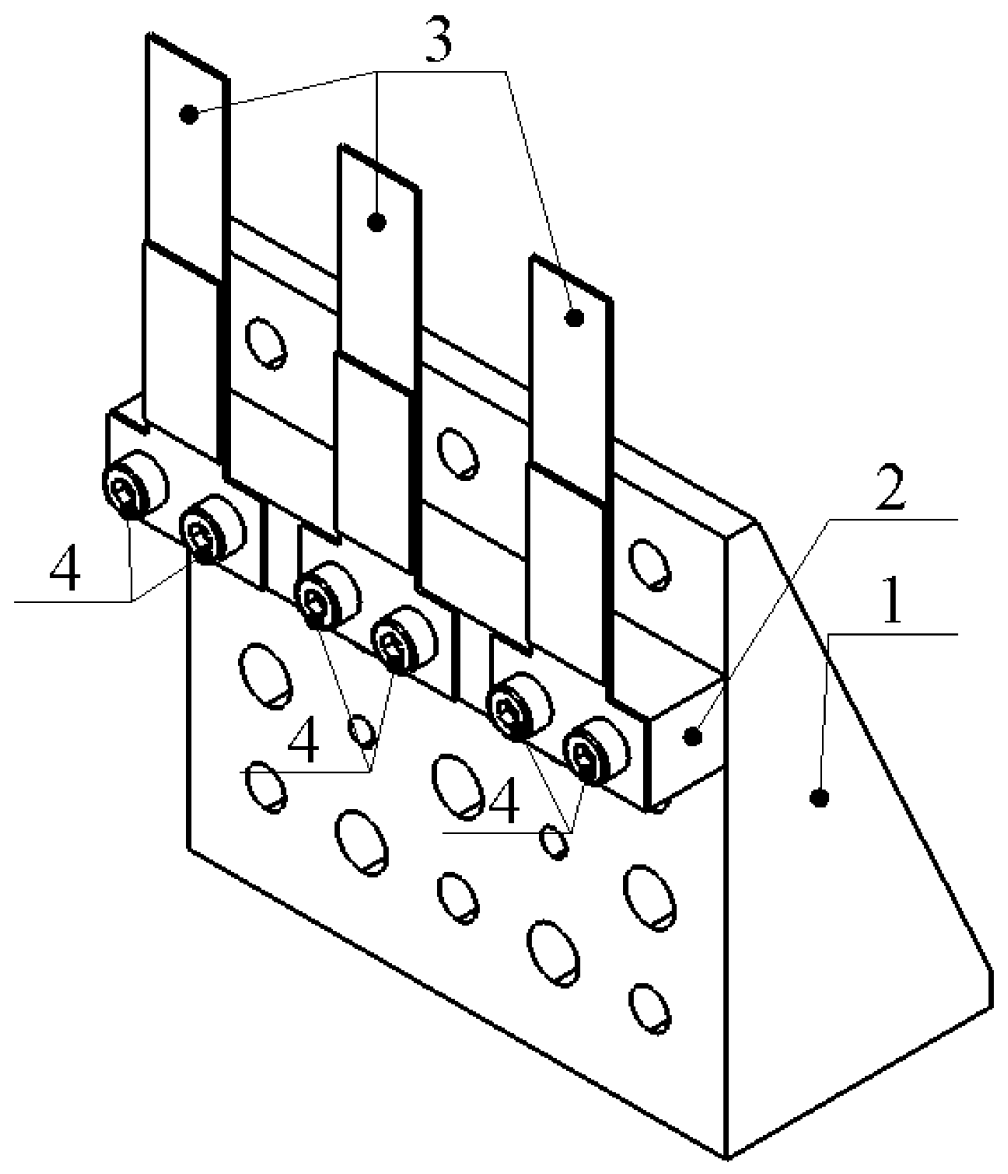
Structural design of clamping system; 1—optical L-shaped bracket; 2—spacing bar; 3—piezoelectric unimorph actuator; 4—clamping bolts.

A dedicated clamping system was developed to ensure repeatable boundary conditions during the experimental investigation. A well-defined clamping configuration is essential for comparing the electromechanical performance of different piezoelectric unimorph actuators, while variations in mechanical fixation may influence resonance frequency, impedance response and displacement amplitude. The clamping structure was based on an L-shaped optical bracket, which served as a rigid support frame for the actuators. A spacing bar was used to define the position and separation of the actuators and to ensure consistent alignment during testing. The structural design of the clamping system is shown in Figure 2.

The clamping system was designed to accommodate three piezoelectric unimorph actuators simultaneously. This arrangement enabled direct comparison of specimens assembled with different bonding materials under identical mechanical and environmental conditions. Each actuator was fixed through the clamping holes using clamping bolts, while the common support structure minimized differences caused by external assembly variations.

The use of a common test stand is particularly important in the present study while the influence of passive-layer material and bonding material is evaluated through changes in resonance frequency, impedance and displacement response. Therefore, the support structure had to ensure stable fixation, repeatable boundary conditions and comparable mechanical loading for all actuators.

## 3. Numerical Investigation of Electromechanical Characteristics of Piezoelectric Unimorph Actuators

Numerical investigations were carried out to evaluate the modal and dynamic behavior of the piezoelectric unimorph actuators and to determine their modal characteristics, impedance–frequency responses and displacement–frequency characteristics at temperatures in the range from 253.15 K to 323.15 K. For this purpose, the numerical model was built using COMSOL Multiphysics 5.6 software. The geometry characteristics of the actuators were set as given in Figure 1 and Table 1, while material characteristics used to build the model are given in Table 2. Boundary conditions of the numerical model were set as well, i.e., the clamping zone of the actuators was fixed rigidly, while a voltage with an amplitude of 80 V_p-p_ was applied to the top electrodes of piezoelectric plates. The bottom electrodes of the piezoelectric plates were set as neutral. The numerical investigations were carried out for two cases, i.e., while the passive part of the unimorph actuator was made from 7075-T6 aluminum and from Ti-6Al-4V titanium. For both cases, the material characteristics of the piezoelectric material were set to PIC 181. On the other hand, geometrical and material characteristics of the bonding material were not included in all numerical models in order to indicate characteristics of actuators without bonding material impact, while its impact will be assessed during experimental investigations.

**Figure 3:**
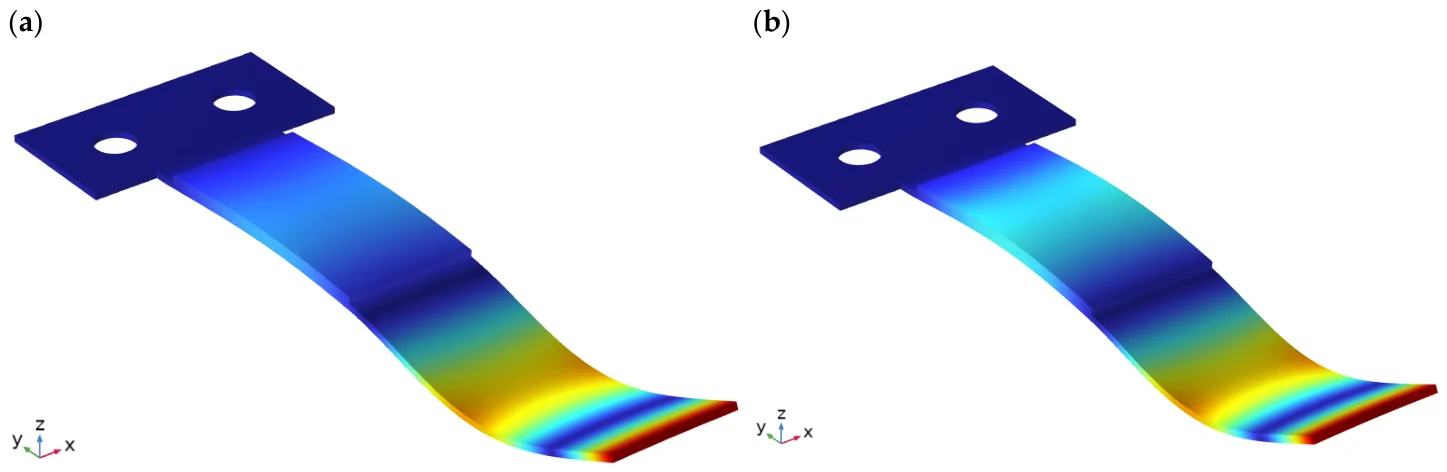
The second, out-of-plane, bending modes of piezoelectric unimorph actuators at 293.15 K; (**a**)—passive layer made of 7075-T6 aluminum with natural frequency of 3339.8 Hz; (**b**)—passive layer made of Ti-6Al-4V titanium with natural frequency of 3405.5 Hz.

**Figure 4:**
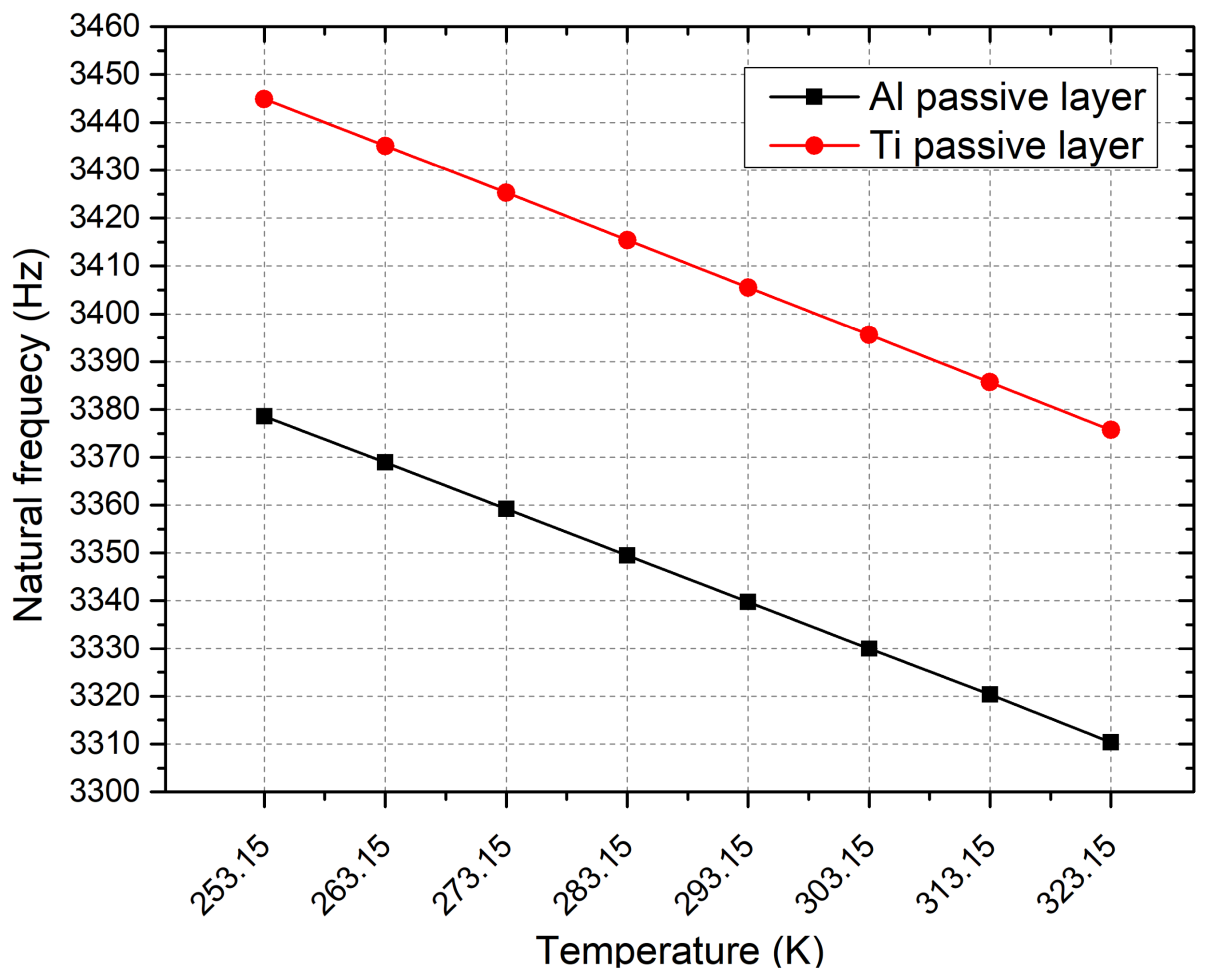
Natural frequencies of the second, out-of-plane, bending mode of piezoelectric unimorph actuators at different environment temperatures.

The first stage of numerical investigation was dedicated to the indication of the second, out-of-plane, bending mode and its natural frequencies of the actuators with passive layers made of aluminum and titanium. The environmental temperature was set to 293.15 K while other boundary conditions were set as described above. The second out-of-plane bending mode was selected as the target mode because it exhibits greater complexity compared to the first bending mode, particularly in terms of the different stress components induced in the actuator, while providing more stable vibration behavior compared to higher-order out-of-plane bending modes. The results of the calculations are given in Figure 3 and Figure 4.

Results of the modal analysis showed that the second, out-of-plane, bending mode of the piezoelectric unimorph actuator with a passive layer made of 7075-T6 aluminum occurs at 3339.8 Hz at a temperature of 293.15 K, while the same vibration mode of the actuator with a passive layer made of Ti-6Al-4V titanium occurs at 3405.5 Hz. This indicates that the actuator with the titanium passive layer has greater dynamic stiffness and therefore exhibits a higher natural frequency of the target vibration mode.

So, in order to indicate drift of natural frequencies of the second, out-of-plane, bending mode while environment temperature changes, the modal analyses of both actuators were performed. Temperature was set from 253.15 K to 323.15 K with an increment step of 10 K. The results of the calculations are given in Figure 4.

As can be found in Figure 4, the natural frequencies of the second, out-of-plane, vibration modes in both cases decrease linearly while temperature increases from 253.15 K to 323.15 K. Also, it can be found that the piezoelectric unimorph actuator with a passive layer made of titanium has higher natural frequencies compared to the actuator with an aluminum-based passive layer, which was initially confirmed by modal analysis at a temperature of 293.15 K.

Taking 293.15 K as the reference temperature, the absolute natural frequency drift of the actuator with an aluminum-based passive layer increases from 9.7 Hz at 283.15 K to 38.8 Hz at 253.15 K. At higher temperatures, the drift increases from 9.8 Hz at 303.15 K to 29.4 Hz at 323.15 K. The maximum absolute drift within the calculated temperature range is 38.8 Hz, which occurred at 253.15 K. The actuator with a titanium passive layer has a similar tendency, i.e., the absolute drift increases from 9.9 Hz at 283.15 K to 39.4 Hz at 253.15 K. At higher temperatures, it increases from 9.9 Hz at 303.15 K to 29.8 Hz at 323.15 K. The maximum absolute drift is 39.4 Hz, also observed at 253.15 K.

The difference in natural frequencies as well as their drift considering temperature mainly results from the different material properties of the passive layers, particularly Young’s modulus and density. Ti-6Al-4V titanium has a higher Young’s modulus than 7075-T6 aluminum, which gives higher stiffness of the unimorph actuator. At the same time, the density difference between these materials also affects the mass distribution of the actuators. The combined effects of stiffness, mass, and their temperature-dependent variations lead to the observed shifts in natural frequency.

**Figure 5:**
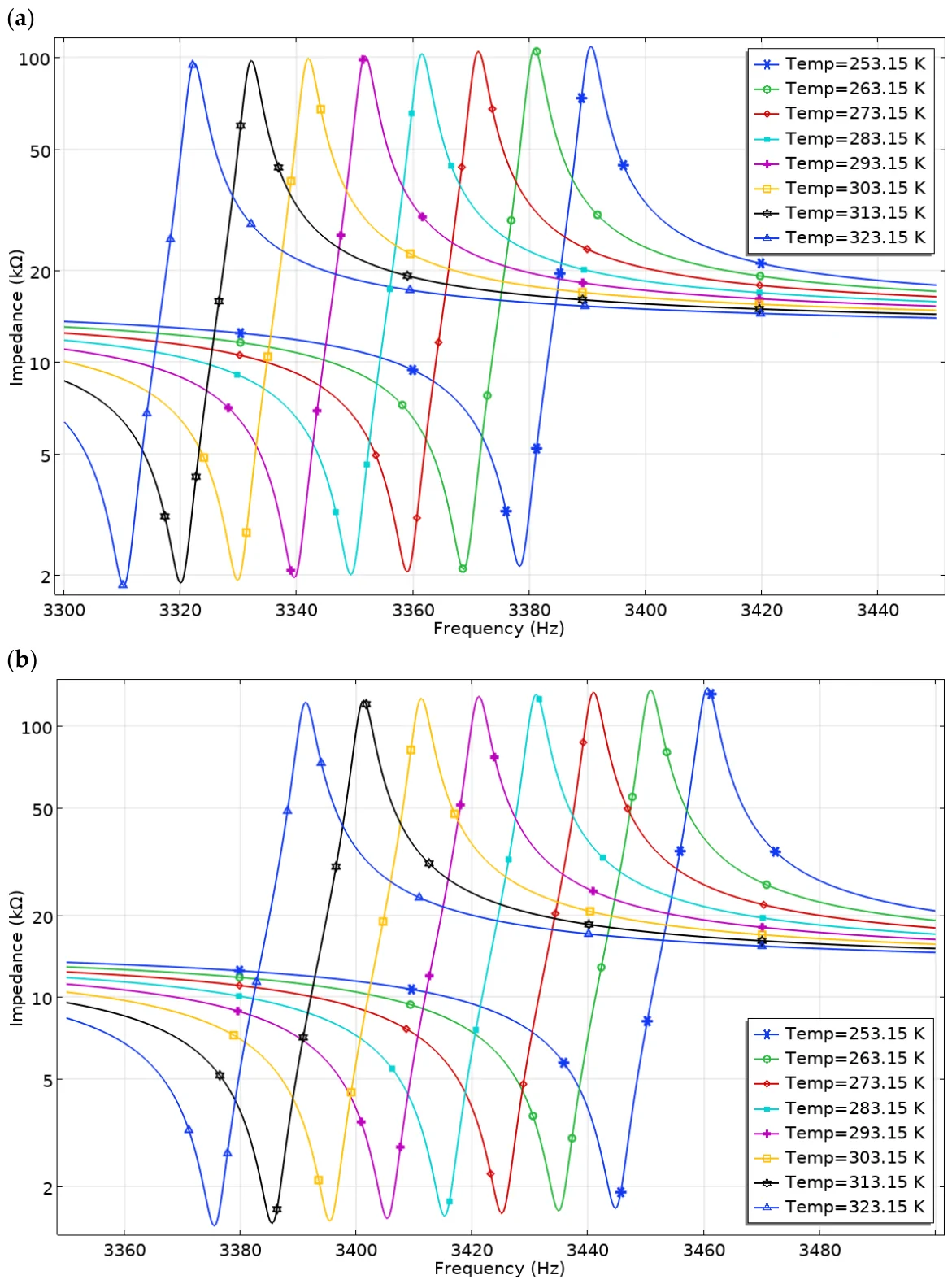
Impedance—frequency characteristics of piezoelectric unimorph actuators; (**a**)—passive layer of piezoelectric unimorph actuator made of aluminum; (**b**)—passive layer of piezoelectric unimorph actuator made of titanium.

The next stage of the numerical investigation was dedicated to the calculation of impedance–frequency characteristics of the actuators with different passive layers. The boundary conditions were set as in the previous case, while the excitation amplitude was set to 80 V_p-p_ for both cases. The frequency ranges were set in accordance with Figure 4, while temperature ranges were set to increase from 253.15 K to 323.15 K with an increment step of 10 K. The results of the calculations are given in Figure 5.

As can be found in Figure 5, the results of calculations show a clear temperature-dependent drift of the resonance frequency for both investigated cases. Taking 293.15 K as the reference temperature, the resonance frequency increased at lower temperatures and decreased at higher temperatures. For the case with an aluminum passive layer, the resonance frequency changed from 3378.30 Hz at 253.15 K to 3310.20 Hz at 323.15 K, corresponding to a total frequency variation of 68.10 Hz. Relative to the reference value of 3339.60 Hz, the drift was +38.70 Hz at 253.15 K and −29.40 Hz at 323.15 K. A similar trend was observed for the titanium passive layer, where the resonance frequency varied from 3444.80 Hz to 3375.50 Hz, giving a total variation of 69.30 Hz. The corresponding drift relative to 293.15 K was +39.40 Hz at 253.15 K and −29.90 Hz at 323.15 K.

The impedance values at resonance also demonstrated a strong dependence on temperature. In both cases, the impedance increased at reduced temperature and decreased with increasing temperature. For the case with an aluminum passive layer, the impedance at resonance increased by 174.12 Ω at 253.15 K, corresponding to +8.89%, and decreased by 116.98 Ω at 323.15 K, corresponding to −5.97%. For the titanium case, the impedance drift increased by 137.90 Ω or +9.05% at 253.15 K and decreased by 92.30 Ω or −6.06% at 323.15 K. This indicates that the electrical response of the piezoelectric actuators is more sensitive to temperature than the resonance frequency.

Overall, both piezoelectric unimorph actuators with aluminum and titanium passive layers exhibit almost linear resonance-frequency drift with temperature. The titanium-based actuator shows a higher absolute resonance frequency, whereas the relative temperature-induced frequency drift is very similar for both cases. The impedance drift is more pronounced than the frequency drift, indicating that temperature has an impact on dielectric, elastic and loss-related material properties and has a significant influence on the electrical characteristics of the piezoelectric system.

**Figure 6:**
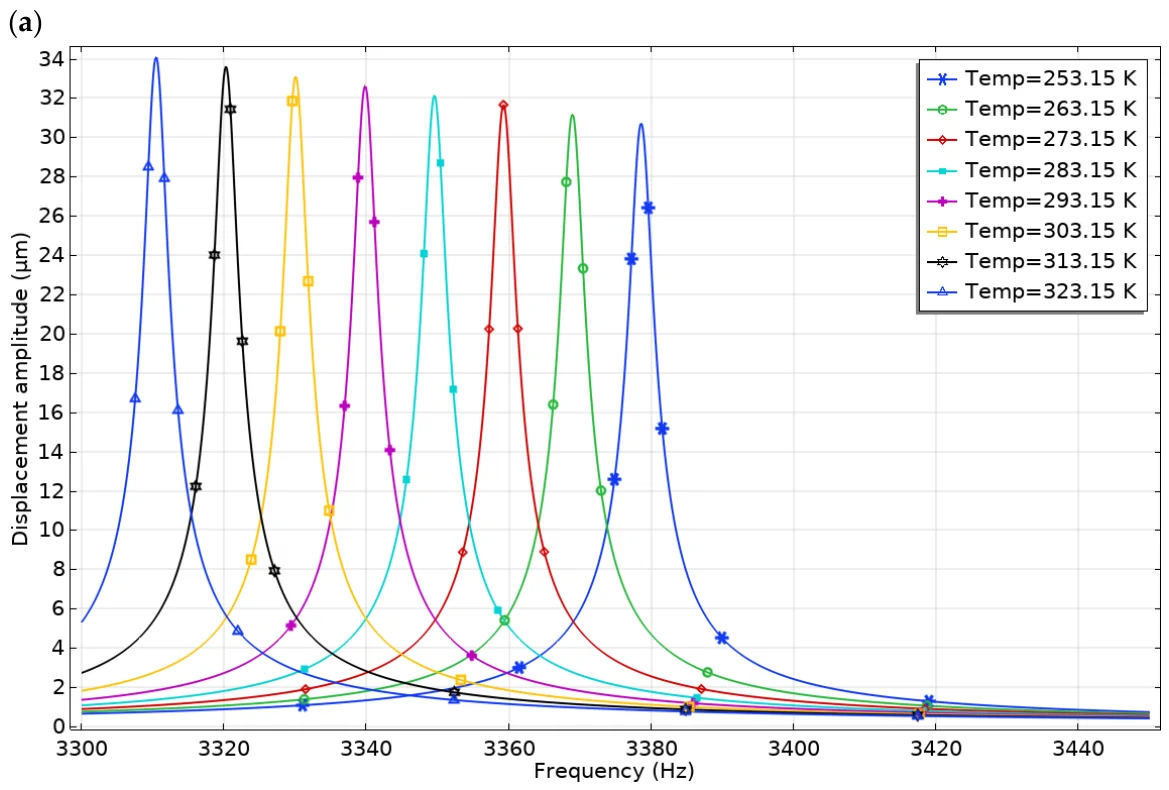

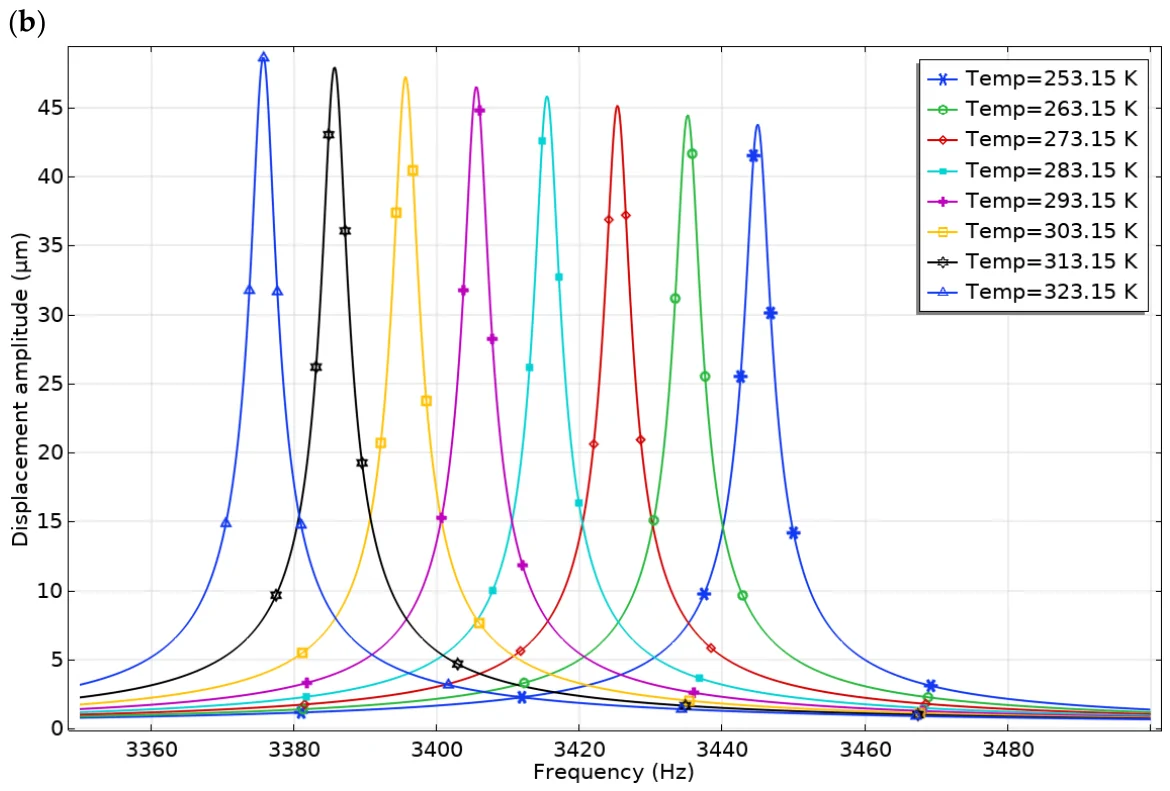
Displacement—frequency characteristics of piezoelectric unimorph actuators; (**a**)—passive layer of piezoelectric unimorph actuator made of aluminum; (**b**)—passive layer of piezoelectric unimorph actuator made of titanium.

The following stage of numerical investigation was dedicated to calculations of displacement-frequency characteristics of piezoelectric unimorph actuators with passive layers made of aluminum and titanium. Mechanical, electrical and thermal boundary conditions were set the same as in the previous case. The results of calculations are given in Figure 6.

The displacement-frequency characteristics (Figure 6) demonstrate temperature-dependent drift for both investigated cases. During analysis of the displacement characteristics of the actuator with aluminum passive layer was found that the displacement amplitude changed from 30.71 µm at 253.15 K to 34.08 µm at 323.15 K. Relative to the reference value of 32.61 µm at 293.15 K, this corresponds to a drift of −1.90 µm at 253.15 K and +1.47 µm at 323.15 K. In relative terms, the displacement amplitude changed from −5.83% to +4.51% over the investigated temperature range.

A similar tendency was indicated during analysis of the actuator with a titanium passive layer. The displacement amplitude increased from 43.80 µm at 253.15 K to 48.66 µm at 323.15 K. With respect to the reference value of 46.53 µm at 293.15 K, the drift was −2.73 µm at 253.15 K and +2.13 µm at 323.15 K. The corresponding relative displacement drift varied from −5.87% to +4.58%. Although the titanium configuration exhibited a higher absolute displacement amplitude through the investigated temperature range, the relative drift was very similar for both cases. The maximum negative drift at 253.15 K was approximately −5.8% for both cases, while the maximum positive drift at 323.15 K was approximately +4.5%. This indicates that temperature affects the displacement characteristics of both piezoelectric actuators.

The increase in displacement amplitude with temperature is associated with temperature-dependent changes in the elastic, dielectric and piezoelectric properties of the piezoelectric material, as well as changes in mechanical losses and structural stiffness. Since the resonance frequencies simultaneously decrease with increasing temperature, the observed increase in displacement amplitude indicates that the dynamic characteristics of the actuators increase at higher temperatures. Therefore, the displacement characteristics are temperature-sensitive and should be considered when defining the operational stability of piezoelectric actuators.

## 4. Experimental Investigation of Electromechanical Characteristics of Piezoelectric Unimorph Actuators

**Figure 7:**
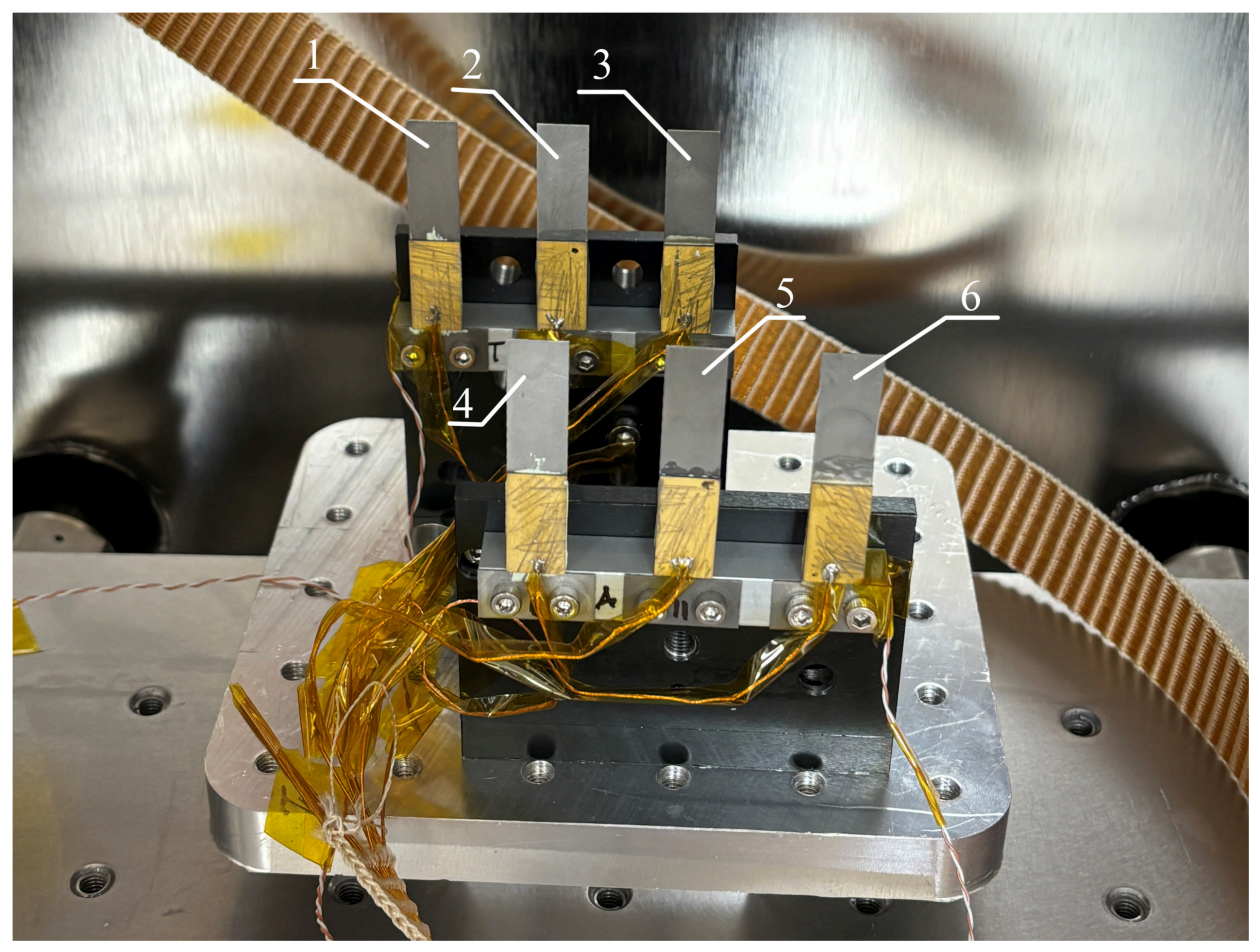
Assembled piezoelectric unimorph actuators; 1—actuator with titanium passive layer and assembled with Bonding material No. 1; 2—actuator with titanium passive layer and assembled with Bonding material No. 2; 3—actuator with titanium passive layer and assembled with Bonding material 3; 4—actuator with aluminum passive layer and assembled with Bonding material No. 1; 5—actuator with aluminum passive layer and assembled with Bonding material No. 2; 6—actuator with aluminum passive layer and assembled with Bonding material No. 3.

In order to perform experimental investigations, prototypes of piezoelectric unimorph actuators were made. Two sets of passive layers made of aluminum 7075-T6 and titanium Ti-6Al-4V with geometrical characteristics as given in Figure 1 and Table 1 were manufactured and then assembled using PIC 181 piezoelectric ceramic plates. The assembly was performed using three different commercial bonding materials, which are suitable for a vacuum environment. A view of prototypes is given in Figure 7.

During the assembly process, particular attention was given to the preparation of the bonding surfaces, since surface condition has a direct influence on adhesion quality and repeatability of the assembled structure. The surfaces of the passive layers were prepared to obtain an average roughness in the range of R_a_ 1.51–2.56 µm, which was selected to promote reliable bonding between the passive layer and the piezoelectric plate. Such surface preparation helps to ensure appropriate mechanical interlocking and stable bond formation during the adhesive joining process. In order to bond the piezoelectric plates to the passive layers, three different bonding methods were used. Characteristics of the bonding materials are given in Table 3.

These adhesives were selected for comparative investigation of their influence on the bonding quality and overall actuator performance. In order to ensure consistent assembly conditions for all specimens, the preload force during bonding was kept identical for all actuators and was set to 20 N. On the other hand, despite preload force and surface roughness, the curing process also plays a significant role in bonding and adhesion quality and, as a result, impacts the performance of the piezoelectric actuators. Therefore, on the basis of the bonding materials manufacturer’s recommendation, the curing was performed in accordance with thermal and time requirements, which are given in Table 4.

**Figure 8:**
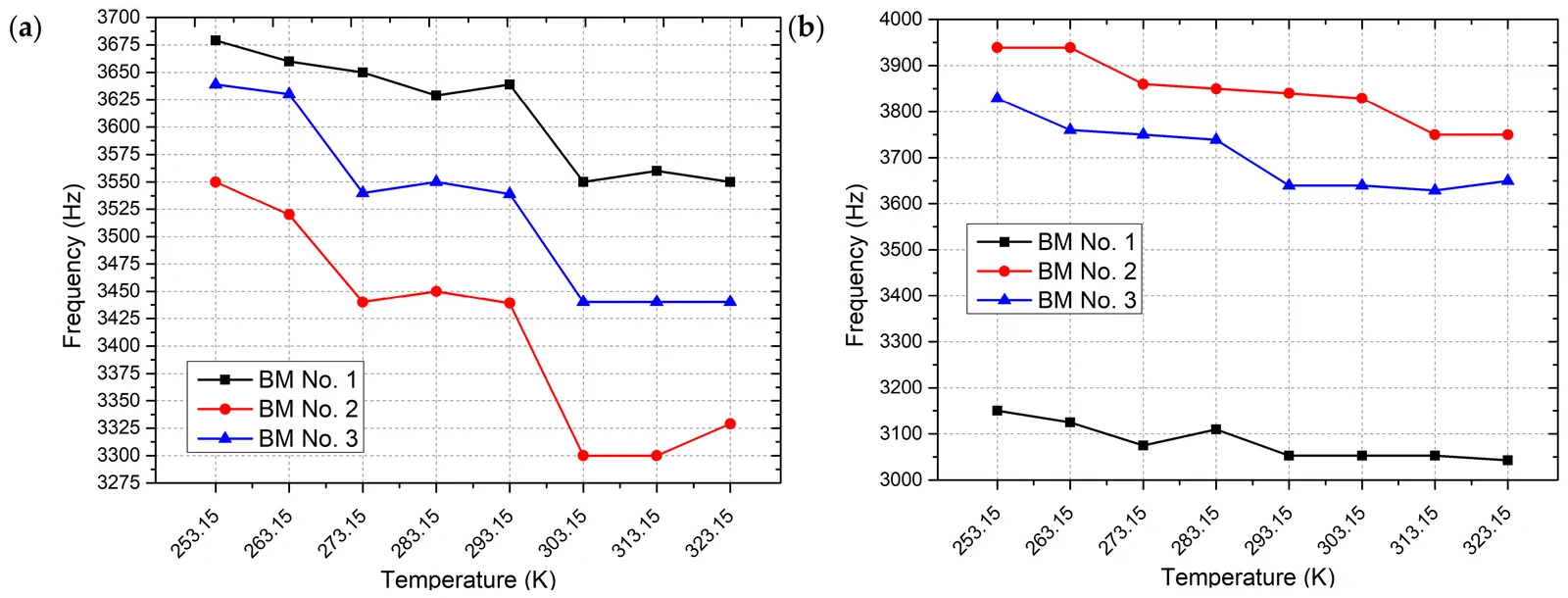
Resonance frequencies of the actuators while different temperatures are set in vacuum; (**a**)—passive layer made of 7075-T6 aluminum; (**b**)—passive layer made of Ti-6Al-4V titanium.

**Figure 9:**
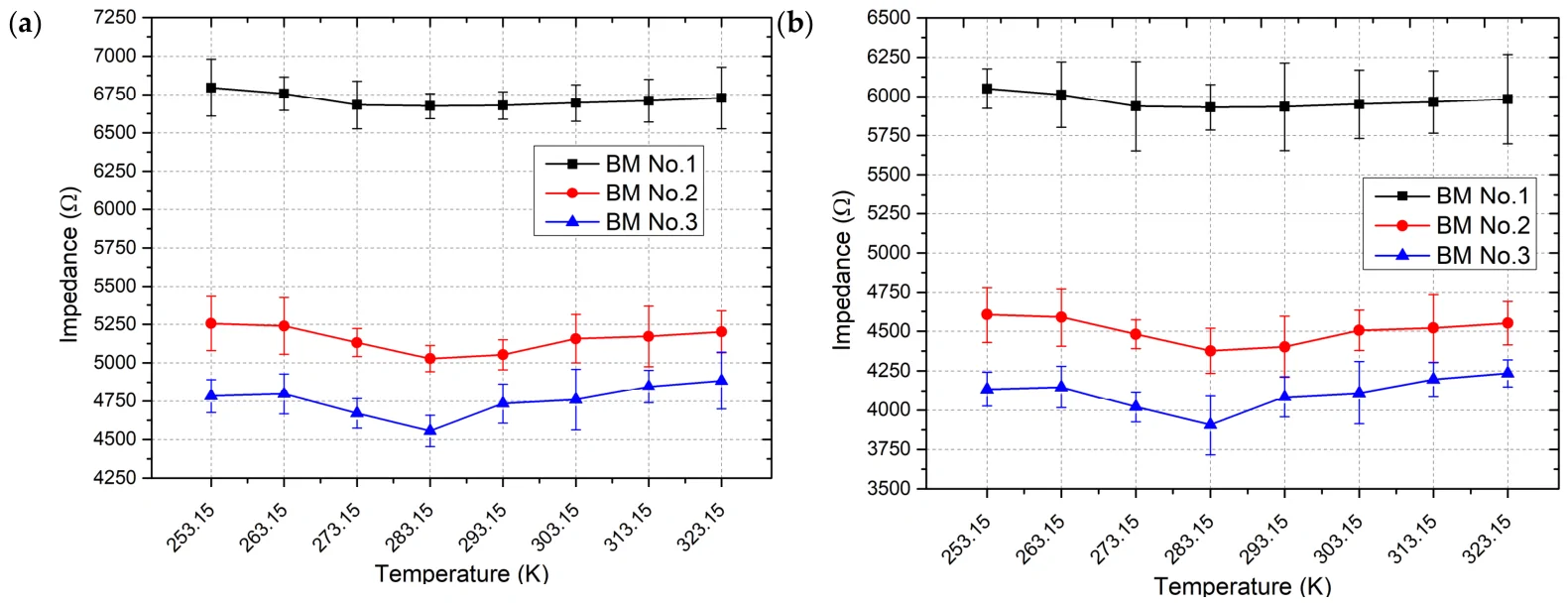
Impedance characteristics of the actuators at resonance while different temperatures are set in vacuum; (**a**)—passive layer made of 7075-T6 aluminum; (**b**)—passive layer made of Ti-6Al-4V titanium.

The first stage of experimental investigation was dedicated to measurements of impedance—frequency characteristics in a vacuum environment (2 × 10^−5^ mbar). For this purpose, both sets of prototypes assembled with different bonding materials and clamped to supports were placed into a thermal vacuum chamber (Telsar TVAC^3^). The measurements of impedance-frequency characteristics were performed by a SinPhase 16667k impedance analyzer. The results of the measurements are given in Figure 8 and Figure 9.

The results in Figure 8 show that, under vacuum and thermal conditions, the resonance frequencies of the second bending mode decrease with increasing temperature for both actuator groups. However, the magnitude and character of this decrease differ between individual specimens and between passive-layer materials. For the actuators with titanium passive layers, the resonance frequencies are generally higher, and their temperature dependence is more gradual. The actuator assembled with BM No. 2 maintains the highest resonance frequency over the entire investigated temperature range, whereas the actuator bonded with BM No. 2 exhibits the lowest values within this group. The observed frequency shift with increasing temperature is moderate, indicating comparatively stable dynamic behavior of the titanium-based actuators under vacuum conditions. On the other hand, the actuators with aluminum passive layers exhibit lower resonance frequencies and a more pronounced decrease at elevated temperatures, particularly above approximately 293.15–303.15 K. The actuators bonded with BM No. 2 and BM No. 3 show a sharper reduction in resonance frequency, suggesting higher sensitivity to thermally induced changes in the mechanical properties of the actuator structure.

The higher resonance frequencies of the actuators with titanium passive layer can be mainly attributed to the higher Young’s modulus and greater structural stiffness of titanium compared with aluminum alloy. The resonance frequencies of the second, out-of- plane, bending mode are governed by the stiffness-to-mass ratio; the stiffer titanium passive layer ensures that the second, out-of-plane, bending mode is at higher frequencies. The greater thermal sensitivity of the actuators with an aluminum passive layer is associated with their lower structural stiffness and the higher sensitivity of the bonded interface to thermally induced stresses.

Differences between individual cantilevers within the same material group can also be attributed to the use of different bonding materials. Since the adhesives differ in stiffness, damping properties and temperature-dependent behavior, they influence the resonance-frequency response of the actuators. Under vacuum conditions, where convective heat-transfer effects are eliminated, the observed resonance-frequency changes are mainly influenced by the combined impact of passive-layer material properties, adhesive characteristics and thermally induced stresses in the bonded actuator structure.

The results given in Figure 9 show that the impedance values at resonance frequencies depend on temperature, passive-layer material and bonding material. For the actuators with titanium passive layers, the actuator bonded with BM No. 3 demonstrates the lowest impedance values over the investigated temperature range. Its impedance decreases from 4134 Ω at 253.15 K to a minimum value of 3906 Ω at 283.15 K, and then increases to 4235 Ω at 323.15 K. This indicates the most favorable electromechanical behavior across the actuators with titanium passive layers. The actuator assembled with BM No. 2 shows intermediate impedance values, ranging from 4377 Ω to 4605 Ω, while the actuator bonded with BM No. 1 has the highest impedance values, ranging from 5930 Ω to 6050 Ω. Therefore, although the actuator assembled with BM No. 1 shows relatively small impedance variation with temperature, its high impedance level indicates less efficient actuator excitation and reduced electromechanical performance.

In the case with aluminum passive layers, the same tendency between actuators was observed. The actuator assembled with BM No. 3 has the lowest impedance values among aluminum-based actuators. Its impedance decreases from 4783 Ω at 253.15 K to 4555 Ω at 283.15 K, and then increases to 4884 Ω at 323.15 K. The actuator bonded with BM No. 2 shows intermediate impedance values, whereas the actuator assembled with BM No. 1 has the highest impedance values over the full investigated temperature range, varying from 6675 Ω to 6795 Ω. This indicates that the actuator with BM No. 2 also provides the most suitable electromechanical response among the actuators with aluminum passive layers.

A comparison of the passive-layer materials shows that the actuators with titanium passive layers generally exhibit lower impedance values than the actuators with aluminum passive layers. This shows that the titanium passive layer provides more stable electromechanical behavior. The lower impedance values of the actuators with titanium passive layers indicate more efficient energy transfer from the piezoelectric layer to the mechanical bending motion of the actuators. Also, the influence of the bonding material is clearly visible in both material groups. In both cases, the actuators bonded with BM No. 3 exhibit the lowest impedance. The actuators bonded with BM No. 2 demonstrate intermediate behavior, whereas the actuators bonded with BM No. 1 show the highest impedance values. Therefore, the impedance results confirm that BM No. 3 provides the most favorable bonding conditions for electromechanical characteristics.

**Figure 10:**
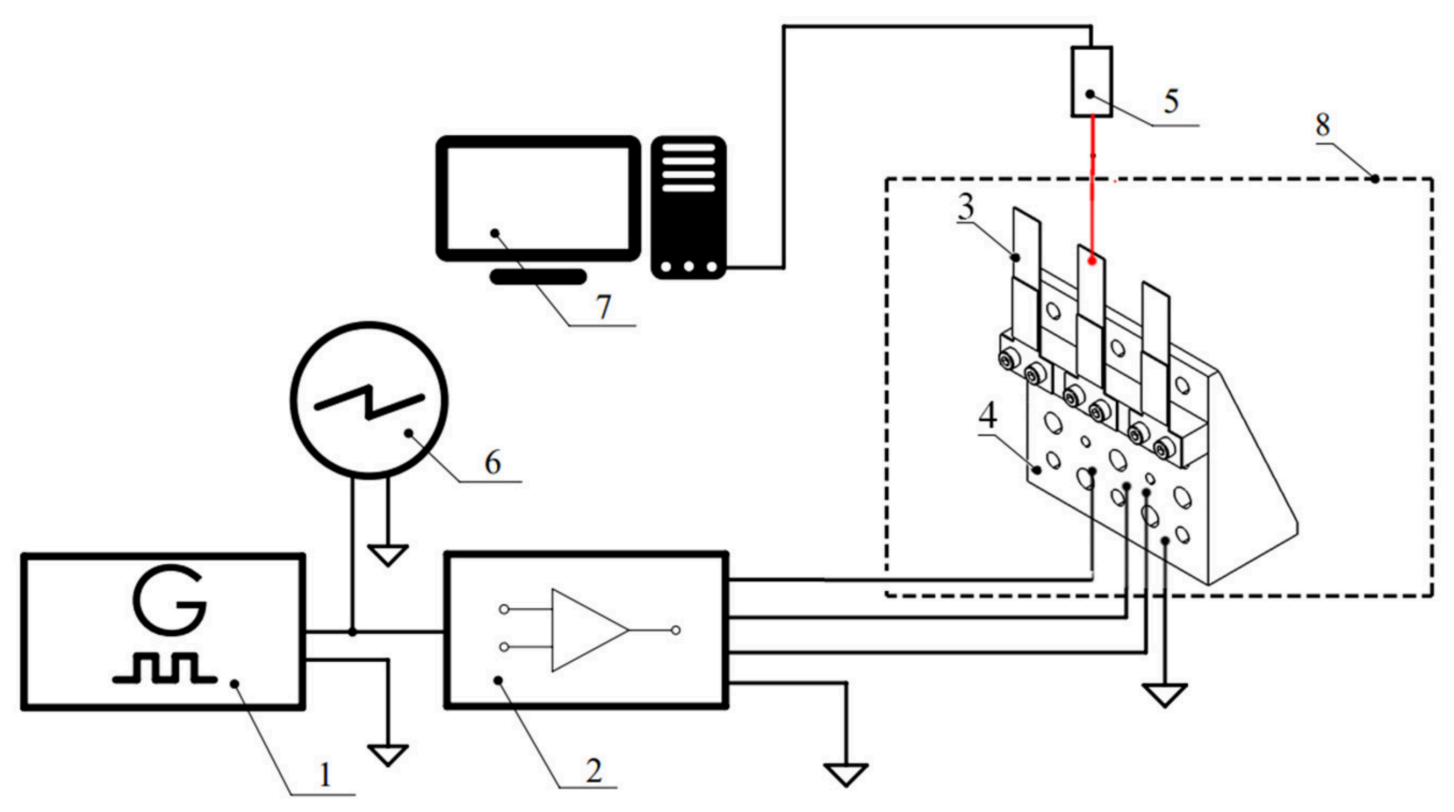
Schematics of experimental setup: 1—a function generator; 2—power amplifier; 3—piezoelectric unimorph actuator; 4—L-shaped support; 5—laser vibrometer; 6—a computer with measuring software; 7—oscilloscope; 8—thermal vacuum chamber.

The next stage of experimental investigation was dedicated to experimental investigations of displacement amplitudes of piezoelectric unimorph actuators composed of aluminum and titanium passive layers and bonded by different bonding materials. For this purpose, an experimental setup was built. A schematic of the experimental setup is given in Figure 10.

**Figure 11:**
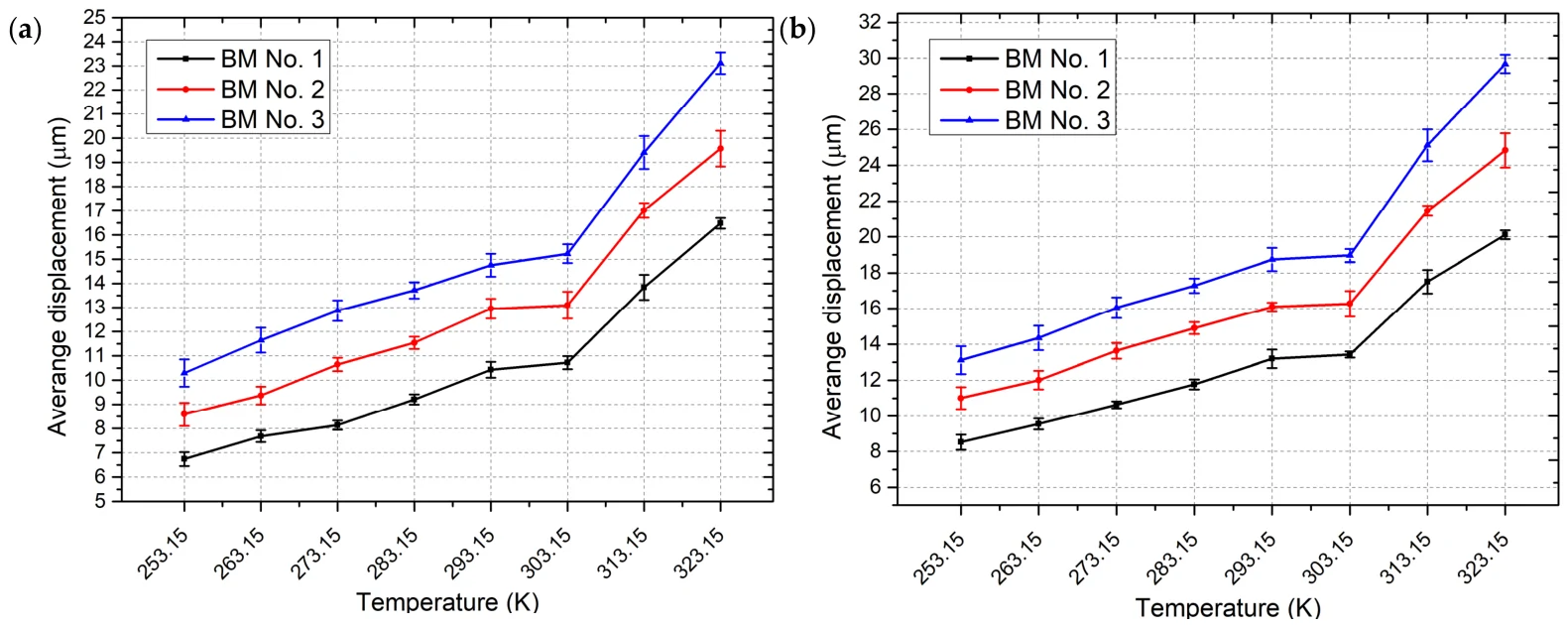
Displacement characteristics of the actuators at resonance while different temperatures are set in vacuum; (**a**)—passive layer made of 7075-T6 aluminum; (**b**)—passive layer made of Ti-6Al-4V titanium.

The experimental setup consisted of a computer, a Tabor Electronics WW5064 function generator, a PiezoDrive PD200x4 power amplifier and a Yokogawa DLM2024 four-channel oscilloscope. The displacement measurements were carried out with a single- point Polytec VibroFlex VFX-I-150 vibrometer. The experiments were conducted inside a Telstar TVAC^3^ thermal vacuum chamber, at a pressure of 2 × 10^−6^ mbar, while the temperature was changed from 253.15 K to 323.15 K with an increment step of 10 K. The out-of-plane displacement amplitudes were measured using a Polytec VFX-I-150 laser vibrometer, which provided non-contact and high-precision measurement of the actuator vibrations while the actuators were driven by an 80 V_p-p_ excitation signal amplitude, whose frequency corresponds to the resonance frequency and environment temperature indicated in Figure 6. The results of the measurements are given in Figure 11.

The results represented in Figure 11 reveal that, under vacuum conditions, the displacement amplitude decreases with decreasing temperature for all investigated actuators. However, the displacement values differ depending on both the passive-layer material and the bonding material. In both actuator groups, the highest displacement amplitudes are observed at 323.15 K, followed by a marked reduction near 273.15 K and a more gradual decrease toward 253.15 K. This confirms that the dynamic response of the unimorph actuators is strongly temperature dependent. A comparison shows that the actuators with titanium passive layers exhibit higher displacement amplitudes over the entire investigated temperature range than the actuators with aluminum passive layers. This indicates that the titanium passive layer provides better dynamic behavior and more efficient strain transfer under the same excitation conditions. The higher stiffness of titanium is considered one of the main reasons for this behavior, since it supports more effective conversion of the strain into bending motion. For the actuators with aluminum passive layers, the highest displacement amplitude was obtained with the actuator assembled with BM No. 3, reaching 23.11 µm at 323.15 K. On the other hand, the lowest displacement amplitude was obtained with the actuator bonded with BM No. 1, reaching 6.74 µm at 253.15 K. In cases with actuators based on titanium passive layers, the highest displacement amplitude was also obtained for the actuator bonded with BM No. 3, reaching 29.67 µm at 323.15 K, whereas the lowest displacement amplitude was observed for the actuator bonded with BM No. 1, reaching 8.52 µm at 253.15 K.

The influence of the bonding material is clearly visible in both cases. Actuators assembled by employing BM No. 1 show the lowest displacement amplitudes within their respective passive layer groups. Actuators bonded with BM No. 2 demonstrate intermediate performance. The highest displacement amplitudes are obtained with actuators assembled using BM No. 3. This indicates that the adhesive layer and its characteristics have a direct influence on the electromechanical response of the unimorph actuators.

It can be noticed that the observed differences can be attributed to variations in adhesive stiffness, damping, compliance and temperature sensitivity. A stiffer and temperature-stable bonding layer improves stress distribution, thereby enhancing the transfer of generated strain to the passive layer. Therefore, the obtained results indicate that the displacement response of the investigated unimorph actuators is governed not only by the passive-layer material, but also by the mechanical and thermal behavior of the bonding material.

## 5. Conclusions

The study investigated the temperature-dependent electromechanical behavior of piezoelectric unimorph actuators under vacuum conditions with a temperature range typical for satellites deployed at low Earth orbit. The numerical and experimental investigations were performed for actuators with two passive-layer materials, i.e., 7075-T6 aluminum and Ti-6Al-4V titanium, over the temperature range of 253.15–323.15 K. The influence of different bonding materials on the resonance frequency, impedance and displacement amplitude was evaluated experimentally. The numerical and experimental results have shown that the electromechanical response of the investigated actuators is governed by the combined influence of temperature, passive-layer material and bonding material. Modal analysis showed that, at 293.15 K, the second out-of-plane bending mode occurred at 3339.8 Hz for the actuator with the 7075-T6 aluminum passive layer and at 3405.5 Hz for the actuator with the Ti-6Al-4V titanium passive layer. This confirms the higher dynamic stiffness of the titanium-based configuration.

Within the investigated temperature range, the numerically determined resonance frequencies decreased almost linearly with increasing temperature. The total resonance-frequency variation was 68.10 Hz for the actuator with an aluminum passive layer and 69.30 Hz for the actuator with a titanium passive layer. Relative to 293.15 K, the impedance at resonance frequencies of the actuator with an aluminum passive layer varied from +8.89% at 253.15 K to −5.97% at 323.15 K, whereas the actuator with a titanium passive layer showed a variation from +9.05% to −6.06%. The displacement-frequency characteristics showed that amplitudes increased with temperature, reaching 34.08 µm and 48.66 µm at 323.15 K for the aluminum- and titanium-based actuators, respectively.

Experimental investigations performed under vacuum conditions confirmed that actuators with Ti-6Al-4V passive layers generally exhibited higher resonance frequencies, lower impedance values and higher displacement amplitudes than actuators with 7075-T6 aluminum passive layers. The bonding material had a pronounced effect on actuator performance. In both actuator groups, BM No. 3 provided the lowest impedance and the highest displacement response. The maximum measured displacement amplitudes reached 23.11 µm for the actuator with an aluminum passive layer and 29.67 µm for the actuator with a titanium passive layer, both at 323.15 K. Therefore, the Ti-6Al-4V passive layer combined with BM No. 3 can be considered the most effective configuration for achieving enhanced electromechanical performance in vacuum for the temperature range typical for satellites deployed in low Earth orbit.

The principal advance of this work is the demonstration that the thermal-vacuum response of piezoelectric unimorph actuators cannot be attributed solely to the piezoceramic or passive-layer material. The bonding interface introduces a substantial additional contribution to resonant impedance and displacement, and its influence persists across the investigated temperature range. By comparing identical actuators manufactured with two passive-layer materials and three bonding materials under the same thermal-vacuum and excitation conditions, the study provides a material-selection framework that is not available from analyses considering the passive layer or bonding interface independently.

## Figures and Tables

**Table 1:** Geometrical characteristics of the actuator.

Parameter	Value, mm	Description
L	55.00	Total length of piezoelectric unimorph actuator
L_Uni_	45.00	Length of unclamped unimorph actuator
L_pzt_	20.00	Length of piezoelectric layer
W_Uni_	10.00	Width of piezoelectric unimorph actuator
W_clamping_	20.00	Width of rectangular clamping zone
W_pos_	5.00	Position of clamping hole
D_clamping_	3.10 mm	Diameter of clamping zone
t_pzt_	0.5 mm	Thickness of piezoelectric layer
t_passive_	0.5 mm	Thickness of passive layer

**Table 2:** Material characteristics used in the numerical model.

Material Properties	PIC 181	7075-T6 Aluminum	Ti-6Al-4V Titanium
Density [kg/m^3^]	7600	2810	4430
Young’s modulus [N/m^2^]	6.3 × 10^10^	7.1 × 10^10^	11.4 × 10^10^
Poisson’s coefficient	–	0.33	0.34
Isotropic structural loss factor	–	0.001	0.001
Relative permittivity (ε_33_^t^/ε_0_)	1300	–	–
Elastic compliance [10^−12^ m^2^/N]	S_11_^E^ = 15.0	–	–
Elastic stiffness [N/m^2^]	C_33_^E^ = 17.0 × 10^10^	–	–
Piezoelectric constant d_33_ [10^−12^ m/V]	289	–	–
Piezoelectric constant d_31_ [10^−12^ m/V]	125	–	–
Coeff. of thermal expansion [1/K]	3 × 10^−6^	2.1 × 10^−5^	8.6 × 10^−6^

**Table 3:** Characteristics of bonding materials.

Property	Unit	Bonding Material (BM)		
		No. 1	No. 2	No. 3
Tensile strength	MPa	41–48	30–35	41–48
Shear strength (Al–Al)	MPa	6.9–8.3	12	4.8–6.2
Elastic modulus	GPa	3.8–4.1	0.34	3.8–4.5
Compressive strength	MPa	165–180	~100	179–193
Shrinkage on cure	%	<0.1	<0.5	<0.1
Hardness	Shore D	85–95	57	85–95
Dielectric constant (1 kHz, 23 °C)	-	4.5	5.5	4.4
Volume resistivity	Ω·cm	>10^15^	1.9 × 10^12^	>10^14^
Stiffness variation (ΔE/E, −40 → +80 °C)	%	<10	>20	<10
Bonding integrity under vibration	-	Excellent	Moderate	Excellent
Viscoelastic loss	-	Moderate	High	Low
Coefficient of thermal expansion (CTE)	×10^−6^ K^−1^	15–18	134	9–12
Thermal conductivity	W/m·K	1.0–1.3	0.39	0.5–0.7
Glass transition temperature (Tg)	°C	~120–130	~65–70	~140
Service temperature range	°C	4 K → +121	−196 → +66	4 K → +149
Outgassing compliance	–	Pass (NASA)	Pass (NASA)	Pass (NASA)

**Table 4:** Curing schedule for different bonding materials.

Bonding Material (BM)	Curing Schedule
No. 1	Stage 1: 12 h at 296.15 K with preload force of 20 N
	Stage 2: 6 h at 353.15 K with preload force of 20 N
No. 2	Stage 1: 24 h at 296.15 K with preload force of 20 N
	Stage 2: 2 h at 339.15 K with preload force of 20 N
No. 3	Stage 1: 24 h at 296.15 K with preload force of 20 N
	Stage 2: 4 h at 363.15 K with preload force of 20 N

## Data Availability

The original contributions presented in this study are included in the article. Further inquiries can be directed to the corresponding author.
